# Economic Evaluation of Screening for Diabetic Retinopathy among Chinese Type 2 Diabetics: A Community-based Study in Kinmen, Taiwan

**DOI:** 10.2188/jea.JE2007439

**Published:** 2008-10-01

**Authors:** Tao-Hsin Tung, Hui-Chuan Shih, Shih-Jen Chen, Pesus Chou, Chi-Ming Liu, Jorn-Hon Liu

**Affiliations:** 1 Cheng Hsin Rehabilitation Medical Center; 2 Faculty of Public Health, School of Medicine, Fu-Jen Catholic University; 3 Department of Nursing, Kaohsiung Armed Forces General Hospital; 4 Department of Ophthalmology, Veterans General Hospital; 5 Community Medicine Research Center & Institute of Public Health, National Yang-Ming University; 6 Faculty of Medicine, School of Medicine, National Yang-Ming University

**Keywords:** Population, Diabetic Retinopathy, Costs and Cost Analysis, Mass Screening, Diabetes Mellitus Type 2

## Abstract

**Background:**

This community-based study conducted in Kinmen aimed to discover whether screening for diabetic retinopathy (DR) among Chinese with type 2 diabetes was economically feasible and clinically effective.

**Methods:**

A total of 971 community-dwelling adults previously diagnosed with type 2 diabetes in 1991-1993 underwent DR screening in 1999-2002 by a panel of ophthalmologists, who used on-site indirect ophthalmoscopy and 45-degree color fundus retinal photographs. Economic evaluation included estimates for cost effectiveness and the cost utility of screening for DR.

**Results:**

For each DR case, screening efficacy and utility decreased, while cost increased with the length of the screening interval. The cost per sight year gained in the annual screening, biennial screening, 3-year screening, 4-year screening, 5-year screening, and control groups were New Taiwan dollars (NT$) 20962, NT$ 24990, NT$ 30847, NT$ 37435, NT$ 44449, and NT$ 83411, respectively. The cost per quality-adjusted life year gained by the annual screening, biennial screening, 3-year screening, 4-year screening, 5-year screening, and control groups were NT$ 21924, NT$ 25319, NT$ 30098, NT$ 35106, NT$ 40037, and NT$ 61542, respectively. Threshold values indicate that the screening programs are highly sensitive to screening cost in the plausible range.

**Conclusion:**

Screening for DR is both medically and economically worthwhile. Annual screening for DR among Chinese with type 2 diabetes should be conducted. Prevention programs aimed at improving eye care for patients with type 2 diabetes result in both substantial federal budgetary savings and highly cost-effective health care.

## INTRODUCTION

Microvascular and macrovascular diseases are the major causes of morbidity and mortality among type 2 diabetics.^1^ In developed countries, diabetic retinopathy (DR) is a major microvascular disease, which is associated with increased visual impairment in type 2 diabetics.^2^ In Taiwan, previous community-based studies have shown the prevalence and annual incidence density of DR to be 15%-45% and 6.62 × 10^-2^/year (95% confidence interval [CI]: 5.36 ×10^-2^-8.06 × 10^-2^/year), respectively.^3^^,^^4^ DR is a major cause of blindness among diabetics. According to the National Health Insurance (NHI) records, in 1997, approximately 970000 patients had type 2 diabetes in Taiwan.5 On the basis of the annual incidence rate described above, a conservative estimate of more than 60000 new cases of DR every year can be made, but NHI records show that only approximately 30000 subjects receive medical care. In other words, many diabetic subjects with DR have not been diagnosed and do not receive appropriate clinical treatment. According to the NHI payment system, panretinal photocoagulation (PRP) is classified as an invasive therapy, and the charges for PRP in New Taiwan dollars (NT$, US$ 1 ≈ NT$ 30 in April 2008) are NT$ 4940 for the first visit and NT$ 2470 for each subsequent visit.^5^ Because diabetes is a chronic condition, establishing a screening strategy to detect DR early and initiate suitable treatment will both reduce medical costs and improve the patients’ quality of life.

If early treatment of DR reduces the incidence or slows the progression of blindness, it might sufficiently reduce treatment costs in later years, which will offset the costs of screening and early treatment. However, most economic studies have not considered the natural history of DR, and may therefore be incorrect in their estimation. Taiwan’s unique medical environment needs to be carefully analyzed with regard to costs and benefits before universal standards are set. Because there have been few well-organized community-based screening programs for DR among patients with type 2 diabetes in Taiwan, this study with long-term follow-up in Kinmen County was conducted to determine the best screening model for DR screening among Taiwanese patients with type 2 diabetes.

## METHODS

### Organization of Diabetic Retinopathy Screening for Type 2 Diabetics

**Figure 1.**
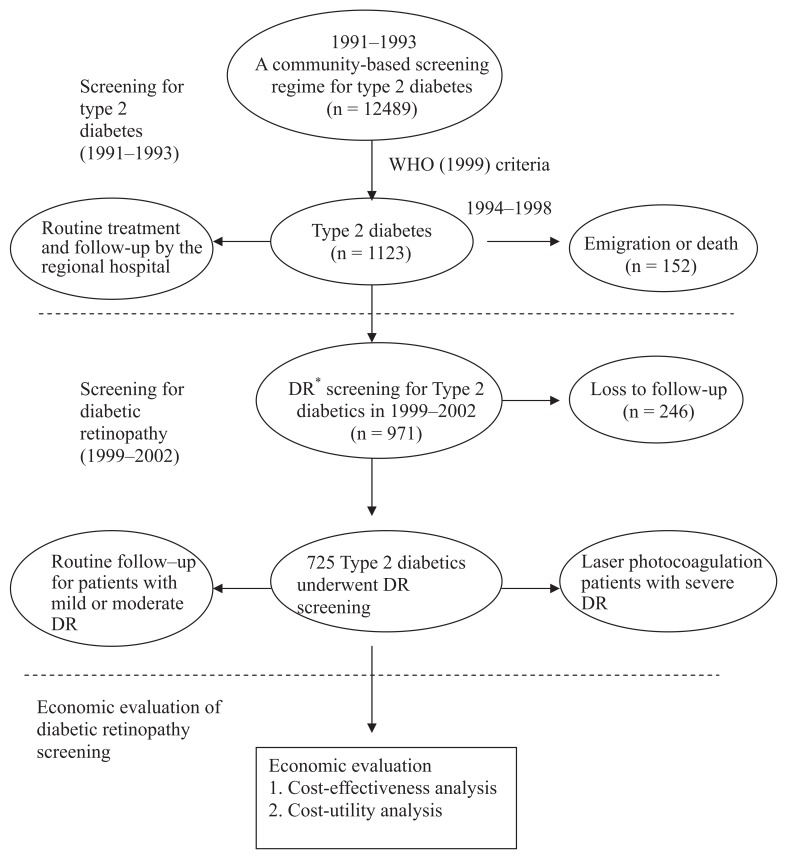
Procedure for economic evaluation of screening for diabetic retinopathy among type 2 diabetics in Kinmen

Figure 1 shows the procedures for economic evaluation of DR screening among community-dwelling patients with type 2 diabetes in the period 1999-2003. The data used in this study were derived from a community-based screening for type 2 diabetes, which targeted subjects aged 30 years or above in Kinmen, Taiwan, between January 1991 and December 1993. Details of the study design and execution have been described in full elsewhere.^6^ Identification of type 2 diabetes was based on the World Health Organization (WHO) 1999 definition,^7^ that is, subjects with a fasting plasma glucose (FPG) level ≥ 126 mg/dL or a 2-h postload glucose concentration ≥ 200 mg/dL were defined as having type 2 diabetes. Subjects with a history of type 2 diabetes and who received medication were defined as known cases. A total of 1123 subjects with type 2 diabetes among subjects aged 30 and above were found from the population survey carried out by the Yang-Ming Crusade, which was organized by the medical students of the National Yang-Ming University, Taipei, Taiwan. The screened diabetic subjects were then referred to the regional hospital for further treatment and routine follow-up. Of the 1123 subjects with type 2 diabetes, 152 emigrated or died between 1994 and 1998. After excluding these subjects, the remaining 971 underwent annual fundus examinations since 1999. A panel for community-based follow-up screening of DR then conducted the screening annually from 1999 through 2002. These 971 participants were invited to undergo eye screening by an invitation letter or call. On the basis of the eye-screening results, different treatment strategies were used, that is, routine follow-up for patients with mild or moderate DR and laser photocoagulation for patients with severe DR. In addition, informed consent was obtained from all the participants before the investigation was initiated. Access to personal records was approved by the hospital’s Human Subjects Review Board at Cheng-Hsin Rehabilitation Medical Center, Taipei, Taiwan.

*: Diabetic retinopathy

We initiated a DR screening program after 6 years of mass screening because Kinmen is an offshore island of Taiwan lacking medical resources. Here, DR screening requires mobilizing manpower and equipment, coordinating between clinical personnel and fieldwork personnel, and transporting equipment to the island. By 1999, a team for DR screening was successfully organized, including 4 well-trained senior ophthalmologists from Veterans General Hospital, Taipei; 4 clinical nurses; and 20 medical students from the Yang-Ming Crusade.

### Screening and Diagnosis for Diabetic Retinopathy

A diagnosis of DR was obtained based on the on-site indirect ophthalmoscopic examination and single-field fundus photographs analyzed later. On-site screening was conducted by senior ophthalmologists who used indirect ophthalmoscopy after pupil dilatation with topical 0.5% mydriacyl.

Graders recorded the findings. Then, one 45-degree color fundus photograph on a Polaroid^®^ 600 film (Polaroid; Nieuw-Vennep, Netherlands) was captured per eye, centered at the macula using a Topcon^®^ fundus camera (TRC-50VT; Tokyo, Japan). The single-field photographs were then printed and filed. Grading of the photographs was performed by 2 well-trained senior ophthalmologists, who began the grading no later than 1 month after the screening. Final grading of DR depended on the summed interpretation of the photographs and the recorded indirect ophthalmoscopic gradings. According to the Diabetic Retinopathy Disease Severity Scale,^8^^,^^9^ DR was classified as follows: no diabetic retinopathy (NDR, no abnormalities), mild non-proliferative diabetic retinopathy (NPDR, only subjects with microaneurysms), moderate NPDR (subjects with more than only microaneurysms but less than severe NPDR), severe NPDR (subjects with any of the following: more than 20 intraretinal hemorrhages in each of the 4 quadrants, definite venous beading in more than 2 quadrants, prominent intraretinal microvascular abnormalities in more than 1 quadrant, and no signs of proliferative diabetic retinopathy), and proliferative diabetic retinopathy (PDR, subjects with 1 or more of the following: neovascularization or vitreous/preretinal hemorrhage). Subjects were classified according to the most severe changes in the worse eye. Blindness was defined by a best corrected acuity of 0.1 (6/60) or worse in the better eye.^10^ Subjects were considered DR cases if they were diagnosed with any type of DR or blindness.

To assure a consistent diagnosis of DR between ophthalmologists, the kappa statistic was used to assess the agreement of interobserver reliability among the study ophthalmologists. A pilot study was performed by randomly selecting 50 subjects with type 2 diabetes other than the study subjects. For interobserver reliability, the kappa value selected for which the diagnosis of DR could be considered to be in good agreement was 0.73 (95% CI: 0.48-0.98).^11^

### Economic Evaluation of Screening for Diabetic Retinopathy

Decision tree analysis is a technique that can be used for selecting an optimal decision by formulating the problem in a tree-structured format, including decision node, chance node, and value node. An expected value for each node is calculated. The best decision is selected on the basis of the expected values. In this study, the economic evaluation tool used for screening of DR among subjects with type 2 diabetes was based on the TreeAge^®^ software (DATA 3.5; Tree-Age Inc., Williamstown, MA), which was used for medical decision analysis using the tree structure and influence diagram approaches.

**Figure 2.**
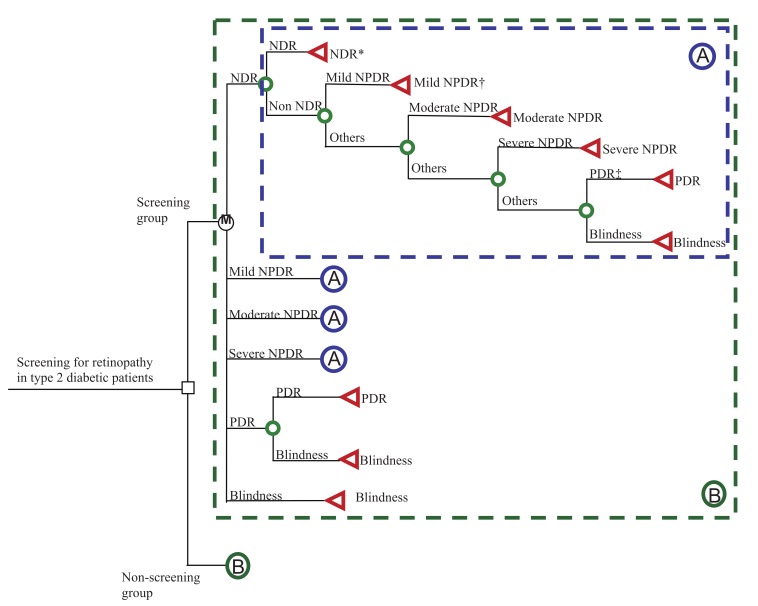
Markov decision model for 2 options, i.e., screening and non-screening for diabetic retinopathy

In this study, a decision analysis using the Markov decision model was constructed to compare different screening regimes for DR with a no-screening group (see Figure 2). The assumption for the no-screening group was that except for eye screening, diabetic patients still received routine medical care until they became blind. According to the theory of stochastic process, the Markov chain model is determined by both the initial state and the transition matrix. The model starts from the decision to screen or not to screen, and the overall expected value is based on the expected values of the end nodes rather than all the nodes. For each decision, there are 6 states for the disease natural history of DR, including NDR, mild NPDR, moderate NPDR, severe NPDR, PDR, and blindness. The initial state distribution is based on the results of the present study. Transition probabilities from one state to another representing the disease natural history of DR were derived from our empirical estimation, that is, the annual transition probabilities from each stage to the next were as follows: mild NPDR to moderate NPDR, 19.4%; moderate NPDR to severe NPDR, 17.4%; severe NPDR to PDR, 29.0%; and PDR to blindness, 21.1%.^12^ For each scenario, we calculated the expected probability of the patients’ aggregate experience that is accumulated in each state during the 10-year follow-up.

* NDR: No diabetic retinopathy.

† NPDR: Non-proliferative diabetic retinopathy

‡ PDR: Proliferative diabetic retinopathy

The costs incurred in the present study include direct and indirect costs. The direct cost include the costs of DR screening, drugs, regular clinic fees, and treatment (for example, laser photocoagulation and surgery). The indirect cost comprised only the productivity loss of the patient because of time taken off work for the treatment. The average time taken off work for the treatment depended on the nature of the profession. All costs are expressed in NT$.

We used a cost-effectiveness analysis (CEA) to compare the cost per sight year gained between the screening and non-screening groups. In order to adjust for the quality of life, a series of utility scores from the utility analysis was assigned for no DR, NPDR, PDR, and blindness.^13^ In brief, the utility evaluation from the time trade-off method was used as a standard procedure with some modification.^14^ The whole scenario was described as follows: “Suppose a situation wherein you could live for 10 years with your current health status. Now, you are given the opportunity to return to a state of perfect health. This opportunity could increase your quality of life but decrease your survival. What is the maximum number of years you would be willing to give up if you could receive this opportunity and have perfect health for the remainder of your life?” The utility value was then calculated by dividing the number of years a subjects was willing to trade in return for an improved life by the estimated number of years of life remaining and subtracting this number from 1.0, that is, utility value = 1.0 - (time traded/time remaining).^14^^,^^15^ The cost-utility analysis (CUA) approach was then used to compare the cost per quality-adjusted life year (QALY) gained between the screening and non-screening groups.

One-way sensitivity analyses were conducted on the individual estimates to assess the impact on costs, effectiveness, and utility of screening for DR. In order to take time preference into account, that is, in order to ensure that the benefits are gained earlier and the costs are incurred later, we discounted all costs and benefits to the present value at 5% annually.

## RESULTS

Table 1 shows the annual direct and indirect costs incurred in the decision analysis of DR screening. Direct costs include screening cost, drug cost, regular clinic fees, and the cost of laser photocoagulation and vitrectomy. Indirect cost represents the lost productivity according to patient’s disease state, which is estimated using the Gross Domestic Product (GDP) value in 2004.

**Table 1.** Cost assumptions, utility value, and transition probabilities in the decision analysis of screening for diabetic retinopathy

Parameter			Value
Annual direct cost (New Taiwan dollars)			
	Screening cost*		2298
	Drug cost^†^		10857
	Regular clinic fees^‡^		509
	Laser photocoagulation^§^		10970
	Vitrectomy^||^		10840
	Total		35474
	
Annual indirect cost (New Taiwan dollars)			
	Gross Domestic Product (GDP)		452168
	
Utility (quality of life) value^13^			
	No diabetic retinopathy (DR)		0.94 ± 0.11
	Non-proliferative diabetic retinopathy (NPDR)		0.87 ± 0.14
	Proliferative diabetic retinopathy (PDR)		0.83 ± 0.09
	Legal blindness		0.81 ± 0.08
	
Annual transition probability (%)^12^			
	No DR	→ Mild NPDR	7.37
	Mild NPDR	→ Moderate NPDR	19.37
	Moderate NPDR	→ Severe NPDR	17.41
	Severe NPDR	→ PDR	28.95
	PDR	→ Legal blindness	21.1

*: Screening cost includes clinician’s fee, vision examination, pupil dilation, slit-lamp contact-mirror funduscopy, funduscopic examination, hemoglobin A1c, simultaneous multichannel autoanalyse-12 test (albumin, alkaline phosphatase, bilirubin, blood urea nitrogen [BUN], calcium, total cholesterol, creatinine, glucose, phosphorus, aspartate aminotransferase [AST, GOT], total protein, and uric acid), and manpower cost.

†: According to the drug usage distribution from the Taiwanese Association of Diabetes Educators (TADE) study in 2004 and the payment of National Health Insurance.

‡: Regular clinic fees includes the clinician’s fee and pharmacist’s fee.

§: Laser photocoagulation cost includes panretinal photocoagulation, 2 fundus color photos, and fluorescein angiography (FAG).

Table 2 shows the results of the 10-year Markov analysis of different DR screening regimens. In each case, screening efficacy and utility decreased, while the cost increased with longer DR screening intervals. After a 10-year follow-up of all screening groups (biennial, 14.2%; 3-year screening, 21.8%; 4-year screening, 28.8%; and 5-year screening, 35.3%) or the non-screening group (59.7%), annual screening showed the lowest probability of blindness (6.6%).

**Table 2.** The results of the 10-year Markov analysis of different screening programs for diabetic retinopathy

Screening strategy	Efficacy*	Utility^†^	Cost (NT$)^‡^	Probability					

				No DR^§^	Mild NPDR^||^	Moderate NPDR	Severe NPDR	PDR^¶^	Blindness
Annual screening	8.2055	7.8458	172007	0.70634	0.09813	0.07035	0.02648	0.03294	0.06576
Biennial screening	7.9071	7.8046	197601	0.64075	0.08952	0.06567	0.02526	0.03713	0.14167
3-year screening	7.5781	7.7667	233761	0.58282	0.08145	0.05984	0.02304	0.03497	0.21790
4-year screening	7.2499	7.7310	271403	0.53017	0.07409	0.05443	0.02096	0.03197	0.28837
5-year screening	6.9336	7.6976	308189	0.48235	0.06741	0.04953	0.01907	0.02910	0.35254

Control group	5.5763	7.5580	465130	0.30055	0.04200	0.03086	0.01188	0.01813	0.59657

*: mean years of sight

†: quality-adjusted life-year

‡: New Taiwan dollar

§: diabetic retinopathy

||: non-proliferative diabetic retinopathy

¶: proliferative diabetic retinopathy

Table 3 shows the results of CEA for different DR screening programs during the 10-year follow-up. Annual screening had the lowest cost and highest effectiveness. The cost per sight year gained (Average Cost-Effectiveness Ratio, ACER) in the annual screening, biennial screening, 3-year screening, 4-year screening, 5-year screening, and control groups were NT$ 20962, NT$ 24990, NT$ 30847, NT$ 37435, NT$ 44449, and NT$ 83411, respectively. Compared with the non-screening group, the screening groups showed better efficacy and less cost. In other words, any screening program was more cost-effective than no screening. Table 3 also shows that after adjustment for utility, annual screening shows a combination of the highest QALY with the lowest cost. The cost per QALY gained (Average Cost-Utility Ratio, ACUR) for annual screening, biennial screening, 3-year screening, 4-year screening, 5-year screening, and control groups were NT$ 21924, NT$ 25319, NT$ 30098, NT$ 35106, NT$ 40037, and NT$ 61542, respectively. Compared with no screening, screening was more effective and cost less. Again, any screening program was more cost-effective than no screening.

**Table 3.** Cost-effectiveness and cost-utility analysis for different screening programs for diabetic retinopathy during the 10-year follow-up

Screening strategy	Cost (NT$)*	Effectiveness (sight years gained)	Cost/ Effectiveness (NT$)	Incremental cost- effectiveness ratio (Compared to control group)	Utility (QALY)^‡^	Cost/Utility (NT$)	Incremental cost-utility ratio (Compared to control group)
Annual screening	172007	8.2055	20962	Dominate^†^	7.8458	21924	Dominate^†^
Biennial screening	197601	7.9071	24990	Dominate^†^	7.8046	25319	Dominate^†^
3-year screening	233761	7.5781	30847	Dominate^†^	7.7667	30098	Dominate^†^
4-year screening	271403	7.2499	37435	Dominate^†^	7.731	35106	Dominate^†^
5-year screening	308189	6.9336	44449	Dominate^†^	7.6976	40037	Dominate^†^

Control group	465130	5.5763	83411	-	7.558	61542	-

*: New Taiwan dollar

†: Any screening program was more cost-effective than no program.

‡: quality-adjusted life-year

Table 4 shows the sensitivity analysis with CEA and CUA for different DR screening regimes. The threshold values indicate that the screening programs are highly sensitive to costs within the plausible range.

**Table 4.** Sensitivity analysis of cost-effectiveness and cost-utility analysis of different screening programs for diabetic retinopathy

Variable	Base case	Range	Threshold of CEA^‡^	Threshold of CUA^§^
Annual screening				
Screening cost (NT$)*	2298	1,000-50,000	33055	34914
Drug cost (NT$)	10857	5,000-50,000	Dominate ^¶^	Dominate ^¶^
Indirect cost (NT$)	113042	0-452,168	1553	Dominate ^¶^
Percentage of laser treatment in PDR^†^ state	0.75	0.1-0.9	Dominate ^¶^	Dominate ^¶^
Percentage of surgical treatment in the state of blindness	0.6	0.1-0.9	Dominate ^¶^	Dominate ^¶^

Biennial screening				
Screening cost (NT$)	2298	1,000-50,000	29221	30814
Drug cost (NT$)	10857	5,000-50,000	Dominate ^¶^	Dominate ^¶^
Indirect cost (NT$)	113042	0-452,168	Dominate ^¶^	Dominate ^¶^
Percentage of laser treatment in PDR state	0.75	0.1-0.9	Dominate ^¶^	Dominate ^¶^
Percentage of surgical treatment in the state of blindness	0.6	0.1-0.9	Dominate ^¶^	Dominate ^¶^

3-year screening				
Screening cost (NT$)	2298	1,000-50,000	25043	26391
Drug cost (NT$)	10857	5,000-50,000	Dominate ^¶^	Dominate ^¶^
Indirect cost (NT$)	113042	0-452,168	Dominate ^¶^	Dominate ^¶^
Percentage of laser treatment in PDR state	0.75	0.1-0.9	Dominate ^¶^	Dominate ^¶^
Percentage of surgical treatment in blindness state	0.6	0.1-0.9	Dominate ^¶^	Dominate ^¶^

4-year screening				
Screening cost (NT$)	2298	1,000-50,000	20902	22020
Drug cost (NT$)	10857	5,000-50,000	Dominate ^¶^	Dominate ^¶^
Indirect cost (NT$)	113042	0-452,168	Dominate ^¶^	Dominate ^¶^
Percentage of laser treatment in PDR state	0.75	0.1-0.9	Dominate ^¶^	Dominate ^¶^
Percentage of surgical treatment in blindness state	0.6	0.1-0.9	Dominate ^¶^	Dominate ^¶^

5-year screening				
Screening cost (NT$)	2298	1,000-50,000	16927	17829
Drug cost (NT$)	10857	5,000-50,000	Dominate ^¶^	Dominate ^¶^
Indirect cost (NT$)	113042	0-452,168	Dominate ^¶^	Dominate ^¶^
Percentage of laser treatment in PDR state	0.75	0.1-0.9	Dominate ^¶^	Dominate ^¶^
Percentage of surgical treatment in blindness state	0.6	0.1-0.9	Dominate ^¶^	Dominate ^¶^

*: New Taiwan dollar

†: proliferative diabetic retinopathy

‡: cost-effectiveness analysis

§: cost-utility analysis

¶: Any screening program was more cost-effective than no program.

## DISCUSSION

Many evidence-based studies have suggested that screening for and treating DR is extremely cost-effective. For those with type 2 diabetes, over a 10-year period, screening saved 67 sight years versus 56 sight years that were saved in the case of no screening, at a cost of US$ 3900 versus US$ 9800 per sight year and US$ 15000 versus US$ 37000 per QALY.^16^ Vijan et al. indicated that in the US population, retinal screening annually versus every other year for patients with type 2 diabetes costs US$ 107510 per QALY gained, while screening every other year versus every third year costs US$ 49760 per QALY gained.^17^ They concluded that annual retinal screening for all patients with type 2 diabetes without previously detected DR may not be cost-effective.^17^ From the health insurer’s perspective, Javitt et al. demonstrated that screening and treatment of eye disease in patients with diabetes cost US$ 3190 per QALY saved.^18^ Polak et al. showed that an additional 1 year of sight gain may cost 1126 Euros in the case of ophthalmological treatments and 50479 Euros in the case of glycemic control treatment: In patients with type 2 diabetes, the duration of blindness falls by 0.48 and 0.13 years, respectively, with an increase in the year of onset of the disease but a decrease in effectiveness.^19^ Prevention programs aimed at improving eye care for patients with type 2 diabetes result in both substantial federal budgetary savings and highly cost-effective health care.^18^

The benefits of DR screening rely on the additional time patients gain, within which they can obtain treatment. However, few community-based studies have attempted to quantify the cost and efficacy of DR screening programs. In type 2 diabetes, the threat of blindness is less severe because DR progresses more slowly than in type 1 diabetes. Although the eye care program saved 21 sight years,^20^ it was less efficient in patients with type 2 diabetes. Nevertheless, for the consideration of both cost and efficacy, many organizations, including the National Committee for Quality Assurance, through Health Employer Data and Information Set (HEDIS) measures, recommends that annual eye examinations be used not only as a general guideline but also as a quality standard in all patients with diabetes.^21^

The present cost-effectiveness and cost-utility analyses show that annual DR screening is the most effective and efficient screening schedule. For early detection, previous epidemiologic studies have indicated that patients with diabetes with mild to moderate NPDR and without macular edema generally require follow-up examination within 6-12 months because as many as 16% of them with mild NPDR can progress to PDR within 4 years.^22^^,^^23^ However, Vijan et al. suggested that in the US population, annual screening offers very little marginal benefit over screening every other year.^17^ For some low-risk groups such as those with good glycemic control, screening every third year may be almost as good as annual screening and is more cost effective.^17^ This study showed different findings because empirical community-based data were not used.^17^ The most aggressive approach would be to recommend annual screening as the safest strategy. In addition, those setting quality of care standards must consider the marginal benefit of frequent fundus examinations.

Although the use of a community-based follow-up study design could reduce selection bias and increase statistical power, the use of primary information and calculation of both direct and indirect costs can help us estimate the true benefit of DR screening more closely than has been possible before. The present study still has certain limitations. First, we did not explicitly consider the sensitivity and specificity of the DR screening tests. Previous studies demonstrated that indirect ophthalmoscopy performed by ophthalmologists has a sensitivity of approximately 85%,^24^ but this may approach 100% with newer slit-lamp biomicroscopic techniques.^25^ Retinal photography, an alternative detection method for DR among diabetic patients, has an overall sensitivity of approximately 85%.^24^ This implies that the accuracy of DR diagnosis could be accepted. Second, although the kappa value for the agreement of interobserver reliability seemed good,^11^ non-differential misclassification bias identification still could have occurred. Third, since type 2 diabetes patients who frequently undergo eye screening are more likely to maintain a suitable blood glucose level and have good prognosis, the effectiveness (sight years gained) of eye screening could be underestimated. In addition, on the basis of the population-based study, the hemoglobin (Hb) A_1c_ level of the diabetic subjects in this study was better than that in clinical investigations. We did not examine how covariates such as the duration of type 2 diabetes or HbA1c level influence the screening efficacy for screening of DR at different intervals. Because severe and very severe NPDR without macular edema is associated with a high risk of progression to PDR, 10-50% of those with type 2 diabetes and this level of NPDR will develop PDR within 1 year.^23^ Further long-term studies should be conducted to clarify whether patients with better glycemic control or in an early stage of DR could benefit from less frequent screening intervals. Fourth, we did not consider indirect costs other than those incurred for screening. The important indirect costs that were overlooked could be a bias for the effectiveness or utility of different screening programs for DR. Finally, it should be noted that the estimates used in this analysis were based on relatively small samples, that is, the DR screening program was conducted on an offshore island of Taiwan lacking medical resources. The aggregate estimates may reflect a reasonable population; however, it does not represent all Chinese with type 2 diabetes, which might question the generalizability of the study. Further study of those inadequately represented is required.

In conclusion, assessing the progression of DR following economic evaluation suggests that screening for DR is worthwhile and that annual screening for Chinese with type 2 diabetes should be recommended.
